# Fruits-Based Critical Nitrogen Dilution Curve for Diagnosing Nitrogen Status in Cotton

**DOI:** 10.3389/fpls.2022.801968

**Published:** 2022-01-28

**Authors:** Weina Feng, Xiaofei Li, Helin Dong, Yukun Qin, Miao Sun, Jingjing Shao, Cangsong Zheng, Pengcheng Li

**Affiliations:** ^1^State Key Laboratory of Cotton Biology, Institute of Cotton Research, Chinese Academy of Agricultural Sciences, Anyang, China; ^2^Cotton Research Institute of Jiangxi Province, Jiujiang, China

**Keywords:** cotton yield, fertilization management, nitrogen nutrition index, petiole nitrate concentration, yield components

## Abstract

Estimating the precise nutritional status of crop nitrogen (N) after flowering period is not only important to predict deficiency but the excess that could be revised by fertilization in future crops. Critical N dilution curves describing the critical N concentration ([N]_c_) in plant tissues during crop growth have been used to estimate the N status of whole plants in cotton. Little is known, however, about the critical N dilution curve for specific plant organs such as cotton fruits. The objective of this study was to verify the feasibility of fruits-based critical N dilution curve as a useful diagnostic tool for diagnosing the N status of cotton crops. A 3-year field experiment was conducted with seven N application rates (0–360 kg N ha^–1^) using the high-yielding cultivars Jimian 228 and Lumian 28, which differ in maturity. The relationship between fruits dry mass (DM) and N concentration ([N]) was analyzed, and a model of [N]_c_ for cotton fruits was constructed and validated. The results showed that fruits [N]_c_ decreased with increasing fruits DM. The critical N dilution curve based on cotton fruits was described by the equation [N]_c_ = 2.49 × DM^–0.12^ (*R*^2^ = 0.649, *P* < 0.0001) across cultivar-years. The N nutrition index (NNI) of the fruits (NNI_f_) with the N dilution curve was significantly related to the NNI of shoot DM, relative yield (RY), and boll density at most sampling dates. For an NNI_f_ of approximately 1, the RY was nearly 95%, while it decreased with a decreasing NNI_f_ below 1. The petiole nitrate-N (NO_3_-N) concentration was also linearly related to the NNI_f_, suggesting that the NO_3_-N concentration in the petiole was a good predictor of the NNI_f_. Therefore, fruits-based critical N dilution curve and the derived NNI_f_ values will serve as a useful diagnostic tool for diagnosing N status in cotton crops.

## Introduction

Cotton is the most important natural textile fiber crop worldwide. Cotton production provides income for approximately 100 million families and its economic impact is estimated to be approximately $500 billion yr^–1^ (Chen et al., 2007; Cotton Research Institute Chinese Academy of Agricultural Sciences [CRI], 2013). Currently, China is the largest producer and consumer of raw cotton in the world. However, the yield is quite low (802 kg ha^–1^) in the Yellow River Valley of China compared with the national average (1720 kg ha^–1^) (United States Department of Agriculture [USDA], 2019; National Bureau of Statistics of China, 2020). One of the main reasons for such a low yield level may be inappropriate crop nutrient management, irrespective of cultivars and climatic variability (Cotton Research Institute Chinese Academy of Agricultural Sciences [CRI], 2013).

Nitrogen (N) is the most common limiting nutrient required by many field crops (Lebauer and Treseder, 2008), including cotton. Currently, China is the largest user of fertilizer N in the world. The production of mineral N fertilizers depends on large amounts of energy from natural gas (oil), a non-renewable resource, and N fertilizer in agricultural production also causes environmental concerns by an unreasonable use (Reis et al., 2016). For example, over-application of N fertilizers in excess of crop requirements is lost to the environment via denitrification, NH_3_ volatilization, surface runoff and leaching (Vitousek et al., 2009; Yan et al., 2014; Reis et al., 2016). There is thus a critical need to optimize the use of N fertilizer to reduce N inputs and to maintain cotton production in sustainable agricultural systems (Zhang et al., 2017; Luo et al., 2018). One way to minimize N fertilization without negatively affecting yield is to match the N supply with crop demand at the correct rate during the growing season (Diacono et al., 2013). Therefore, it is important to develop crop N status diagnostic tools to improve N fertilizer management and achieve sustainable agricultural development goals in China and worldwide.

The N nutrition status of crops can be diagnosed by plant analysis. One of the most frequently used plant N diagnostic methods is based on the determination of critical plant N concentration ([N]_c_) (Plénet and Lemaire, 1999; Debaeke et al., 2012; Ata-Ul-Karim et al., 2014; Lemaire and Gastal, 2018; Giletto et al., 2020). The minimum plant N concentration ([N]) required to achieve its maximum growth rate at a given amount of biomass accumulation, time, and field situation is referred to as [N]_c_ (Greenwood et al., 1990). This concept is based on the premise that an allometric relationship exists between nutrient concentration and biomass accumulation through a dilution process (Plénet and Lemaire, 1999). The critical N concentration can thereafter be used to calculate the N nutrition index (NNI) by dividing the actual [N] in the plant biomass by the [N]_c_ (Lemaire et al., 2008a; Ata-Ul-Karim et al., 2013; Zhao et al., 2016b; Lv et al., 2020).

N critical dilution curves based on the whole plant basis have been established for a range of crops (e.g., wheat, rice, maize, oilseed rape, sunflower, and potato; Debaeke et al., 2012; Ata-Ul-Karim et al., 2013; Yao et al., 2014; Weymann et al., 2017; Giletto et al., 2020; Rodríguez et al., 2020; Zhao et al., 2020), defining scenarios of luxury (N excess), sufficiency and deficiency for plant N status, but only three peer-reviewed studies have been conducted for cotton N critical dilution curve (see Chen et al., 2021 for a bibliometric analysis). The determinations of the critical N dilution curves of cotton have been based on whole plants related to specific cultivars and environmental conditions (Xue et al., 2007; Wang et al., 2012; Ma et al., 2019). These critical N dilution curves differ between species with contrasting genotypes, environments and management combinations (Flénet et al., 2006; Lemaire et al., 2008b; Ata-Ul-Karim et al., 2013; Du et al., 2020; Ciampitti et al., 2021), indicating inter- and intra-species dissimilarities. Moreover, N deficiencies can alter biomass allocation among plant organs and thus the shape of the dilution curves may vary depending on which plant organs are included (Weymann et al., 2017; Giletto et al., 2020; Lv et al., 2020). While the concept of critical N dilution curves to specific plant organs is similar to the whole plant basis, critical N dilution curves for specific plant organs have not yet been determined for cotton, which could be useful for assessing cotton N nutrition status.

The use of specific organs for the diagnosis of N must be sensitive to changes in N in the soil and that absorbed by the plant during cultivation, which allows for correction of N content and productivity. Although the leaf is the most frequently used in plant N diagnosis (Yao et al., 2014; Zhao et al., 2017), the theory of critical N dilution has recently been used to develop critical N dilution curves based on plant reproductive organs, such as wheat spikes, maize ears (Zhao et al., 2020) and potato tubers (Giletto et al., 2020; Lv et al., 2020). N dynamic of the reproductive organ may be more relevant with the formation of yield (Zhao et al., 2020). These findings offer an opportunity to diagnose plant N status for crop-specific N fertilization management during crop post-anthesis period (Zhao et al., 2020). Cotton plants are unique because they are perennial with an indeterminate growth habit, and consequently, the vegetable and reproductive phases can last up to 90 days in parallel (Cotton Research Institute Chinese Academy of Agricultural Sciences [CRI], 2013). The flowering and boll-setting stages are the recommended moment for N analysis and diagnosis because of high nutrient demand by cotton plants. During these stages, for example, the uptake of N is approximately 60–62% of the entire growth period (Zhang and Dong, 2020). Moreover, fruits N uptake and production have a close correlation with final yield (Li et al., 2020). Hence, the accuracy of fruits N status after the flowering period is of prime significance for cotton yield improvement.

Nutrient diagnostic tools should allow easy measurement and interpretation. The direct measurement of plant [N] can offer sufficient knowledge of the limiting and non-limiting factors governing N demand, yet it is expensive, time-consuming and labor-intensive, which considerably limits its adaptation in a large range of situations (Lemaire et al., 2008a; Ata-Ul-Karim et al., 2014). Numerous studies have demonstrated the importance of leaf chlorophyll meter readings as an indirect indicator of plant N status in various crops (Wu et al., 1998; Arregui et al., 2006; Ziadi et al., 2008a; Zhao et al., 2016b). However, chlorophyll meter readings may be highly affected by many external factors other than N, such as environment, position, disease and cultivars (Lemaire et al., 2008a). Due to the relatively more robust and accurate response of chlorophyll meters to N deficiency, the nitrate-N (NO_3_-N) concentration in sap could be an alternative to evaluate N nutrition status (Wu et al., 2007).

Here, a 3-year field experiment with seven N application rates and two cultivars was conducted to evaluate crop N nutrition status using a critical N dilution curve based on cotton fruits for diagnosing the N status of cotton crops. The objectives of this study were: (1) to determine the critical N dilution curve based on cotton fruits for two cultivars; (2) to quantify the critical fruits NNI and its relationship with yield and yield components; and (3) to explore the use of petiole NO_3_-N concentration determined in the fully expanded functional leaves to predict the fruits NNI in cotton.

## Materials and Methods

### Study Site

**FIGURE 1:**
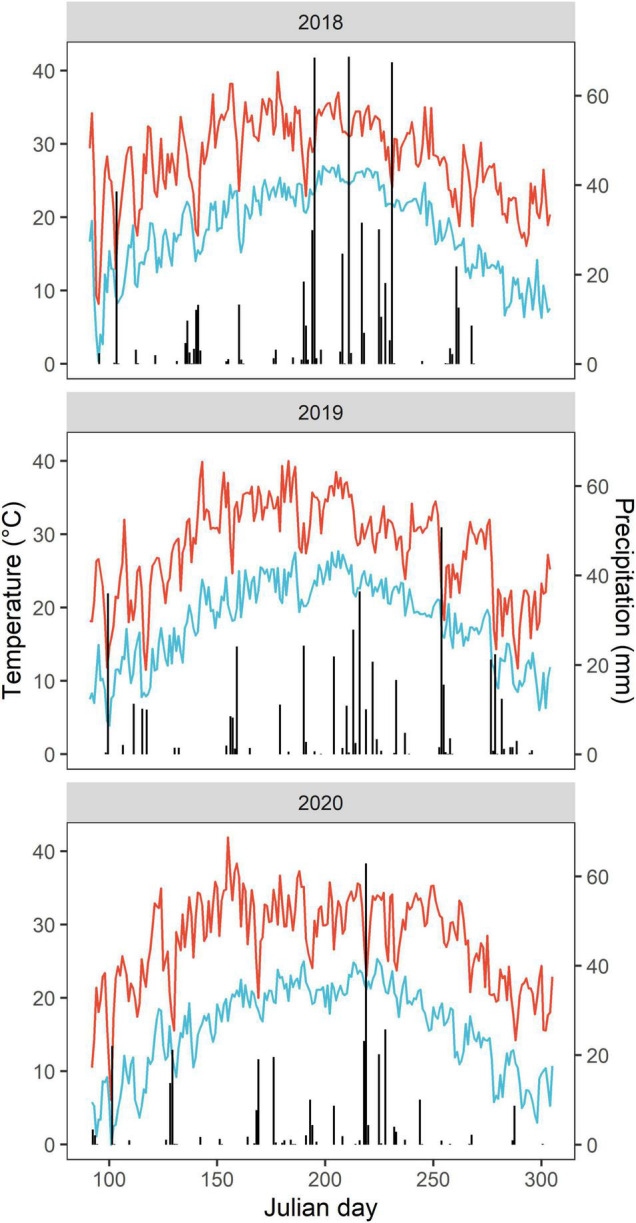
Daily air temperature and precipitation during the cotton growing season from April to October in 2018–2020. The evolution of daily maximum and minimum temperature values are presented as continuous red and blue lines, respectively. The vertical bars depict daily precipitation.

This study was conducted in 2018–2020 at an experimental station (36°13′N, 114°35′W) in Anyang County, Henan Province, China. The site has a typical semi-humid climate with an annual average air temperature of 14.2°C and precipitation of 553.8 mm (1973–2017) (Li et al., 2020). In 2018, 2019, and 2020, annual growing season precipitation was 569.3, 454.1, and 317.5 mm, respectively, and the respective mean growing season temperatures were 22.6, 22.6, and 21.6°C (Figure 1). The soil type is classified as Inceptisols (USDA Soil Taxonomy). The topsoil (0–20 cm) of the experimental field soil is a sandy loam, with a pH (H_2_O) of 8.03, organic matter of 13.8 g kg^–1^, total nitrogen (N) of 0.9 g kg^–1^, Olsen phosphorus (P) of 17.9 mg kg^–1^ and available potassium (K) of 168.4 mg kg^–1^ at the beginning of the experiment.

### Experimental Design and Crop Management

The experiment was a split-plot design with three replicates. The same layout was maintained in the three consecutive years. The main plot factors were seven N application rates (0, 60, 120, 180, 240, 300, and 360 kg N ha^–1^). The subplot factors were two cotton cultivars (Jimian 228 and Lumian 28). Jimian 228 and Lumian 28 were used for their middle and mid-early maturity characteristics, with sprout-to-harvest times of approximately 130 and 125 days, respectively, and they are the main high-yielding cultivars grown in areas of the Yellow River Valley. Each plot was 28.8 m^2^ in area, 3.2 m wide and 9 m long, and included four rows (north-south orientation). Adjacent plots were separated by a 15-cm-high ridge to avoid contamination caused by N dynamics between the treatments. The planting density was 6.0 plants m^–2^ in rows spaced 80 cm apart.

Planting of cotton seeds took place on 28, 30, and 21 April in 2018, 2019, and 2020, respectively. All plots were overseeded and then manually thinned to the desired density at the squaring stage. Cotton plants absorbed about 58% of fertilizer N at 18–20 days after first bloom (Yang et al., 2013), thus allocating more fertilizer N to the later growth stages is of importance to enhance nutrient use efficiency and cotton yield (Yang et al., 2011; Geng et al., 2015). As such, two fifths of the N fertilizer (urea, 46% N) was evenly broadcasted and incorporated into soil at sowing, while the remaining N was applied with irrigation at the early flowering stage to avoid N losses (e.g., N volatilization). Flood irrigation was adopted according to the local farmers’ practice. In addition, P and K were applied as basal fertilizers at 90 kg P_2_O_5_ ha^–1^ and 120 kg K_2_O ha^–1^ in the form of triple superphosphate (44% P_2_O_5_) and potassium sulfate (50% K_2_O), respectively. Other field management practices, such as pest and weed control, were conducted following local practices and remained uniform and normal for all treatments. After harvest, cotton straws for each plot were wholly removed from the field, leaving the soil bare till sowing the next year.

### Plant Sampling and Measurements

Due to variation in maturity of the two cultivars, phenological events (plant emergence, squaring, flowering, and boll opening) were not synchronized but had little difference (Supplementary Table 1). In this study, therefore, sampling started only when 50% of observed plants in each plot reached flowering using the middle maturity cultivar Jimian 228 as a reference. Plant samples were collected at 0, 15, 30, 45, and 60 days after flowering (DAF) in 2018; 0, 17, 44, 60, and 81 DAF in 2019; and 35 and 60 DAF in 2020. Note that two samplings in 2020 were made to validate critical fruits dilution curve. For each sampling, three plants were randomly selected from the middle rows in each plot to avoid border effects. The samples were partitioned into leaves (including petioles), stems, squares, flowers and bolls. All samples were placed in an oven at 120°C for 30 min and then further dried at 80°C until a constant weight was reached. The dry mass (DM) of each plant organ was determined. Dried plant samples were finally ground to a fine powder, after which [N] was determined using the micro-Kjeldahl distillation method (Bao, 2005). Shoot N uptake was obtained from the sum of N in each organ, which was estimated from the product of DM and [N]. Six petiole samples from the fully expanded functional leaves (i.e., the 4th leaf from the apex) on the main stem were collected at each plant sampling date per plot in 2019. The petioles were placed into an ice box immediately after removal and transported to the laboratory for the measurement of nitrate-N (NO_3_-N) concentration using Reflectoquant^®^ (RQ flex^@^ 10 plus, Merck KGaA, Darmstadt, Germany).

### Cotton Yield and Yield Components

We presented cotton yield and yield components in 2018 and 2019. The seedcotton was handpicked from the entire area of each plot on 12 September and 30 October in 2018 and 27 October in 2019. In 2019, only once cotton harvest was made, as the number of opening bolls was lower as a result of bad weather in September (heavy precipitation and low temperature, Figure 1). In both years, we also considered large bolls that opened only after sun drying when determining the final yield. The seedcotton was weighed after sun-drying. Prior to harvest, ten plants were randomly tagged in each plot to record observations on the number of open bolls per plant. For each harvest, all open bolls of the ten plants were collected in each plot, and dried to determine boll weight (seedcotton weight per boll). Lint percentage was calculated as the ratio of lint weight after ginning to seedcotton weight before ginning. The seedcotton yield was multiplied by the lint percentage to estimate the lint yield. In this study, lint yield was used as a proxy for cotton yield, as cotton production is mainly conducted to obtain cotton fibers. Shoot DM was determined as the seedcotton plus stalk yield.

### Data Analysis

#### Critical Nitrogen Dilution Curve for Cotton Fruits

The critical fruits N dilution curve was determined by a negative power function according to the methodology proposed by Justes et al. (1994). Briefly, the data from each sampling occasion were categorized into two groups, namely, (i) N limiting, where an increasing N application rate resulted in a significant response in fruits DM, and (ii) N non-limiting, where an additional N application rate did not lead to a further increase in fruits DM. The [N]_c_ was determined by using the intersection points of the oblique (simple linear regression of N limiting points) and vertical lines (mean values of N non-limiting points) at each sampling time for Jimian 228 and Lumian 28, respectively.


(1) $${\left[N\right]}_{c}=a\times DM^{-b}$$

Where [N]_c_ and DM are the critical N concentration (%) and dry mass (t ha^–1^) of fruits, respectively. Parameter a is [N]_c_ for fruits when fruits DM is exactly 1 t ha^–1^. Parameter b is a dilution coefficient, which describes the decrease in [N] associated with fruits DM increase. The datasets in 2018 and 2019 were used to determine [N]_c_ and establish fruits-based critical N dilution curve. The critical fruits N dilution curve was validated using two sampling datasets in 2020. The preceding formula was also used to calculate the critical N dilution curve for cotton stems and leaves.

#### Nitrogen Nutrition Index Based on Cotton Fruits

To characterize N status, fruits N nutrition index (NNI_f_) was calculated as the ratio of the actual N concentration ([N]) to the critical N concentration ([N]_c_) of fruits (Lemaire and Gastal, 1997):


(2) $$NNI_{f}=\frac{fruits\left[N\right]}{fruits{\left[N\right]}_{c}}$$

Where NNI_f_ values lower than 1 indicate an N deficiency in fruits, values close to 1 indicate that N status is non-limiting for fruits development, and values greater than 1 indicate luxury N accumulation in fruits. The preceding formula was also used to calculate the N nutrition index of the shoot (NNI_sh_).

#### Relative Yield

The RY was calculated as follows (Ata-Ul-Karim et al., 2017):


(3) $$RY=\frac{Y_{i}}{Y_{max}}$$

Where Y_i_ is the lint yield of each plot, and Y_max_ is the maximum lint yield of each cultivar in each year.

### Statistical Analysis

Shoot DM, fruits DM, [N] and petiole NO_3_-N concentrations for each sampling date, along with lint yield, RY and yield components at harvest were subjected to linear mixed effects models with N rate, cultivar and year set as fixed effects and N rate nested within replicate treated as a random effect. In the analysis of petiole NO_3_-N concentration, year was excluded because data were from a single year. Where significant, differences between the means were compared using Tukey’s honestly significant difference (HSD) test at *P* < 0.05.

Non-linear regression analyses were used to examine the relationship between stems, leaves and fruits DM and stems, leaves and fruits [N]. The differences in two N dilution curves between cultivars or years were tested using analysis of covariance after data were log-transformed. We also used simple linear regression to investigate the relationships of NNI_sh_, N rate, RY, boll density, boll weight, and petiole NO_3_-N concentration with the NNI_f_. All statistical analyses were performed in R version 3.6.2 (R Core Team, 2019), with the “nlme” package for the linear mixed effects model (Pinheiro et al., 2020) and the “basicTrendline” package for non-linear and linear regression (Mei and Yu, 2018).

## Results

### Cotton Fruits Dry Mass, Shoot Dry Mass, and the Corresponding Nitrogen Concentration

Overall, N fertilization affected fruits and shoot DM in 80 and 95% of the sampling dates across cultivar-years, respectively. Fruits and shoot DM increased over the growing season, with values ranging from 0.10 to 8.52 t ha^–1^ for fruits and from 1.36 to 15.70 t ha^–1^ for shoot across sampling dates and cultivar-years (Supplementary Tables 2, 3).

In general, fruits and shoot [N] were significantly affected by N fertilization in 45 and 55% of sampling dates across cultivar-years, respectively. As expected, N concentrations decreased during the growing season. Across sampling dates and cultivar-years, fruits [N] ranged from 1.55 to 3.71%, while shoot [N] ranged from 1.38 to 2.77% (Supplementary Tables 2, 3).

### Lint Yield, Relative Yield, and Yield Components

Lint yield and yield components are shown in Table 1. Lint yield and yield components were considerably affected by the N rate and cultivar, but not by their interactions, indicating consistency of the N effect over cultivars and vice versa. Lint yield, boll density and boll weight changed little between N fertilizer rates except when no N was included, and no significant increase above 60 kg N ha^–1^ was observed. Jimian 228 showed a higher lint yield and lint percentage than Lumian 28, with a significant difference between the 2 years. The averaged greatest lint yield and lint percentage was 1725 kg ha^–1^ and 40.2% for Jimian 228, and 1655 kg ha^–1^ and 38.8% for Lumian 28 across 2 years, respectively. Similar results were also observed for RY (Supplementary Figure 1).

**TABLE 1:** Lint yield, boll density, boll weight, and lint percentage in response to year (Y), N application rate (N), and cultivar (C).

Treatments	Lint yield	Boll density	Boll weight	Lint percentage
	kg ha^–1^	bolls m^–2^	g boll^–1^	%
0	1487 b	62.1 c	5.51 b	38.6 a
60	1586 ab	65.6 bc	5.78 ab	38.9 a
120	1603 ab	69.0 ab	5.85 ab	38.9 a
180	1661 a	70.2 ab	6.08 a	39.1 a
240	1684 a	72.1 a	6.18 a	38.8 a
300	1651 a	71.2 ab	5.98 ab	38.8 a
360	1676 a	69.8 ab	5.89 ab	39.0 a
Tukey’s HSD	130.9	6.22	0.55	2.29
Jimian 228	1663 a	67.9 a	5.95 a	39.6 a
Lumian 28	1579 b	69.3 a	5.85 a	38.1 b
Tukey’s HSD	49.5	2.53	0.21	0.71
2018	1588 b	66.1 b	6.10 a	38.0 b
2019	1654 a	71.0 a	5.70 b	39.7 a
Tukey’s HSD	50.8	2.31	0.19	0.68
**ANOVA**				
Y	0.0006	<0.0001	<0.0001	<0.0001
N	0.0185	0.0061	0.0178	0.9848
C	<0.0001	0.1639	0.2344	0.0001
Y × N	0.3112	0.6139	0.0132	0.6869
Y × C	0.1788	0.0804	0.4860	0.3620
N × C	0.8995	0.9882	0.7329	0.5474
Y × N × C	0.4961	0.9894	0.4452	0.8391

*Shown are the means of the three replicate plots and the value of Tukey’s HSD error bar.*

*Different letters indicate significant differences using Tukey’s HSD test at P < 0.05.*

### The Critical Nitrogen Concentration and Nitrogen Dilution Curves in Fruits, Stems, and Leaves

**FIGURE 2:**
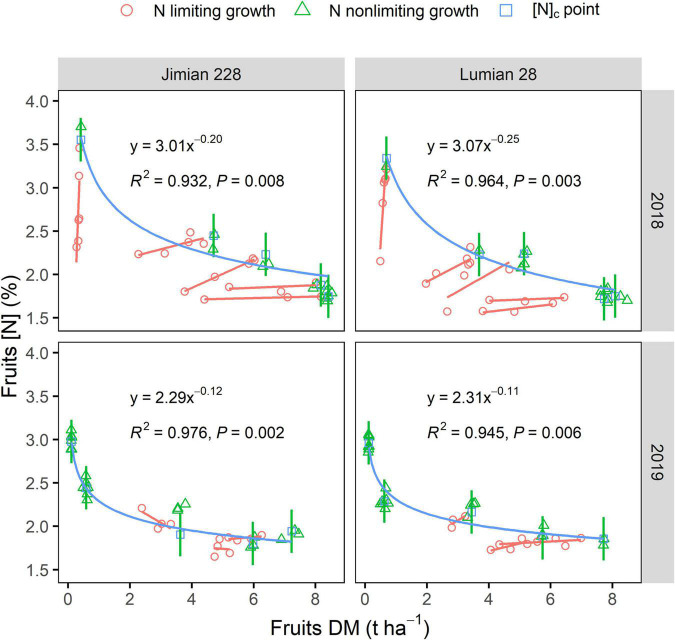
Critical fruits N dilution curves for Jimian 228 and Lumian 28 in 2018 and 2019. DM, dry mass; [N], N concentration and [N]_c_, critical N concentration. Data show means of the three replicate plots. The red lines represent simple linear regression of N limiting growth points at each sampling date. The green lines represent the mean values of fruits dry mass by N non-limiting growth points at each sampling date. The blue lines represent the fitted critical N curves for cotton fruits using [N]_c_ points. Two curves were not significantly different between cultivars in each year (*P* = 0.3910 in 2018; *P* = 0.6605 in 2019) and were significantly different between years for Lumian 28 (*P* = 0.0052) based on ANCOVA. The calculation for the critical N dilution point is shown in the section “Materials and Methods.”

Fruits [N]_c_ showed a gradually declining trend and the decrease was more rapid for the lower N fertilizer rates corresponding to the lower DM values (Figure 2). Fruits [N]_c_ decreased from a maximum of 3.21% to a minimum of 1.86% while the corresponding fruits DM increased from a minimum of 0.33 t ha^–1^ to a maximum of 7.80 t ha^–1^ across cultivar-years. Similar results were also observed for stems and leaves [N]_c_ (Supplementary Figure 2).

**FIGURE 3:**
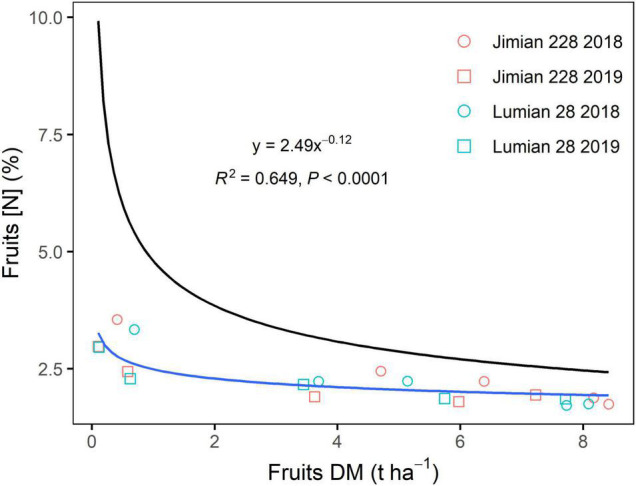
The overall critical fruits N dilution curve across cultivar-years. DM, dry mass and [N], N concentration. Points shown for the mean values of the three replicate plots. The equation and fit statistics (*R*^2^ and *P*-value) are indicated for the relationship. Additionally, the general critical N curve ([N]_c_ = 4.80 × DM^–0.34^) is presented for C3 crops based on plant biomass (the black line).

The decline in fruits [N]_c_ was closely related to fruits DM regardless of the climatic conditions of the year or cultivar, which was described by a negative power function (Figure 2). The N dilution curves between the two cultivars were not significantly different (*P* > 0.05). Therefore, a unique dilution curve across cultivar-years was fitted as follows: [N]_c_ = 2.49 × DM^–0.12^ (*R*^2^ = 0.649, *P* < 0.0001). However, the critical N dilution curves for stems and leaves were not well determined by a negative power function in some cultivar-years cases (Supplementary Figure 2). The explanatory powers of the stems and leaves N dilution curve model (*R*^2^) were also weaker (Figure 2 and Supplementary Figure 2). Moreover, fruits specific N dilution followed a similar trend as that in the case of the entire plant level, but it was a notably lower than the general curve for C3 crops (Figure 3).

**FIGURE 4:**
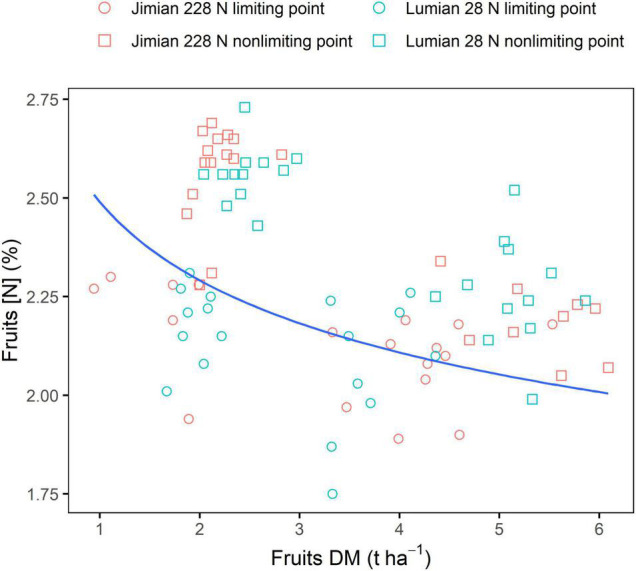
Validation of critical fruits dilution curve using independent datasets from two cotton cultivars in the 2020 cropping season. DM, dry mass; and [N], N concentration. Points shown for the values of the three replicate plots at each sampling date. The data for fruits DM and [N] in 2020 are shown in Supplementary Table 4.

The datasets in 2020 were used to test the accuracy of the critical fruits N dilution curve determined with the 2018 and 2019 datasets (Figure 4 and Supplementary Table 4). The fruits N dilution curve could effectively discriminate between N limiting and N non-limiting situations within the range for which it was established. Most data points from N limiting points were located under the curve, while those from N non-limiting points were close to and above the curve.

### Nitrogen Nutrition Status

**FIGURE 5:**
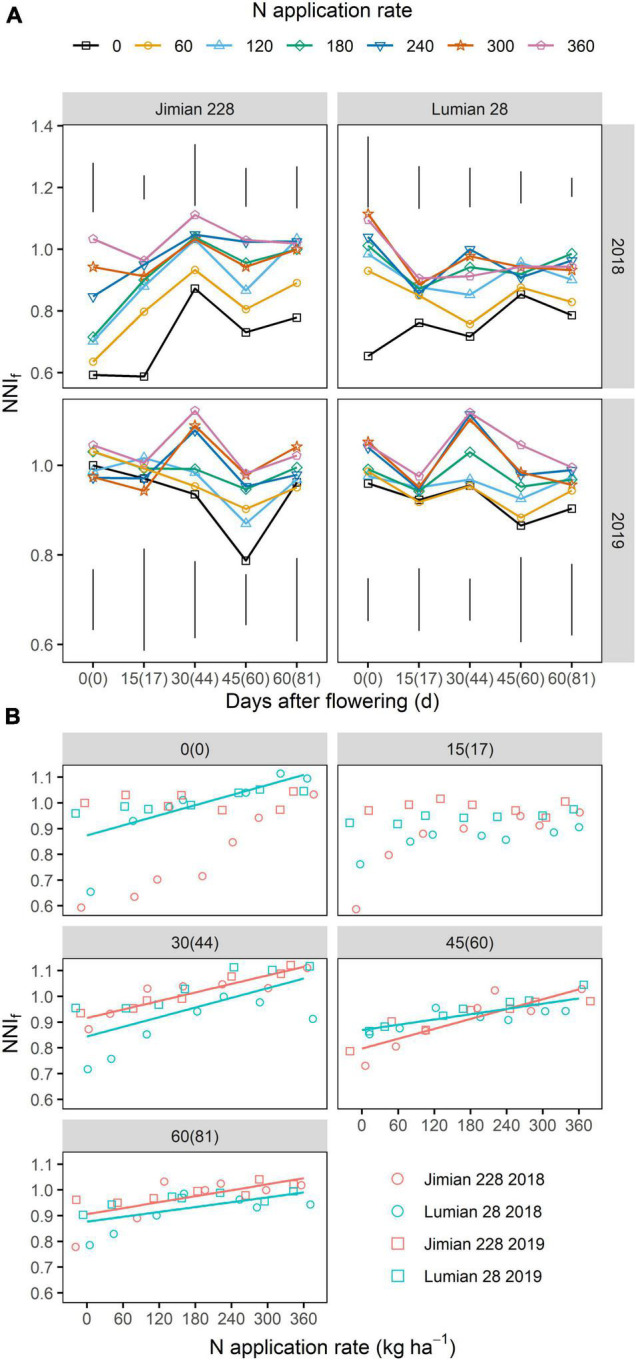
The nitrogen nutrition index of cotton fruits (NNI_f_) after flowering **(A)** and its relationship with N application rate **(B)**. The numbers without and with brackets are the days after flowering corresponding to sampling dates in 2018 and 2019, respectively. **(A)** The vertical bars indicate Tukey’s HSD error bars at each sampling date. For clarity purposes, standard error at each sampling date is not shown in the graphs. ANOVA table is shown in Supplementary Table 5. **(B)** Points shown for the mean values of the three replicate plots. The red and blue solid lines represent significant linear relationships (*P* < 0.05) for Jimian 228 and Lumian 28 across 2 years, respectively; non-significant relationships are not shown (*P* > 0.05).

From the dilution curve developed for fruits DM, the NNI_f_ was calculated for all sampling dates. The NNI_f_ changed little between sampling dates. Across sampling dates, N rates and years, NNI_f_ values ranged from 0.822 to 1.033 (average 0.994) for Jimian 228, and 0.838–0.998 (average 0.943) for Lumian 28, respectively (Figures 5A,B). In both cultivars, moreover, N rates positively affected the NNI_f_ at most sampling dates (Figures 5A,B), and the highest NNI_f_ value was recorded with the highest N application. The NNI_f_ was not affected (*P* > 0.05) by cultivar on average (Supplementary Table 5).

**FIGURE 6:**
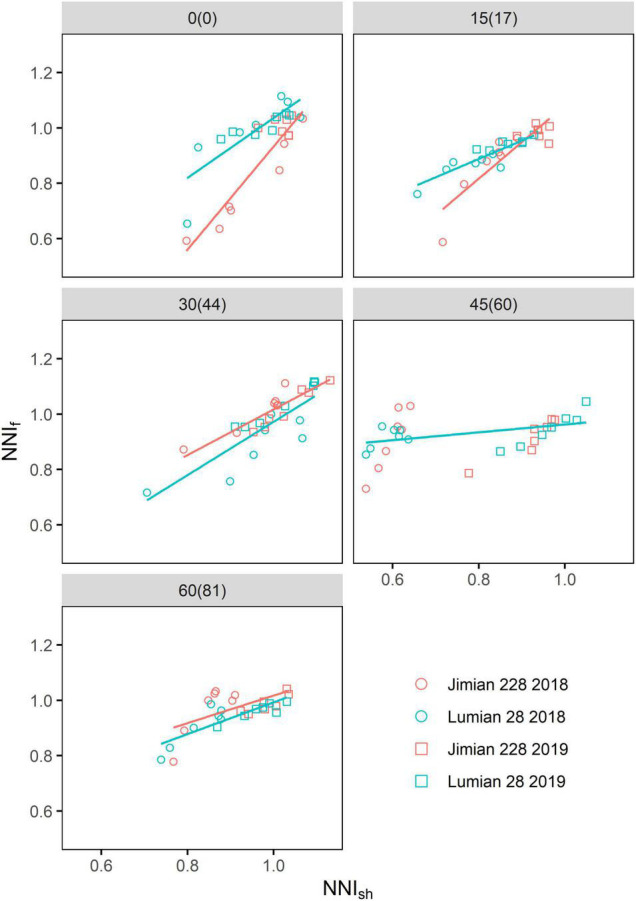
The relationships between the shoot nitrogen nutrition index (NNI_sh_) and fruits nitrogen nutrition index (NNI_f_) for the two cotton cultivars at each sampling date. The NNI_f_ (NNI_sh_) is calculated as the ratio between the actual fruits (shoot) N concentration and the critical fruits (shoot) N concentration. Points shown for the mean values of the three replicate plots. The values without and with brackets are the days after flowering corresponding to different sampling dates in 2018 and 2019, respectively. The red and blue solid lines represent significant linear relationships (*P* < 0.05) for Jimian 228 and Lumian 28 across the 2 years, respectively; non-significant relationships are not shown (*P* > 0.05).

The NNI_f_ values were highly related to the NNI_sh_ for both cultivars in most cases, with the exception of Jimian 228 at the fourth sampling after the flowering period (Figure 6), indicating the strong synchrony between fruits and shoot N status. When the NNI_sh_ was equal to one, the average NNI_f_ was 0.968 and 0.963 for Jimian 228 and Lumian 28 across sampling dates and years, respectively.

### Relationships of Nitrogen Nutrition Index of Fruits With Relative Yield and Yield Components

**FIGURE 7:**
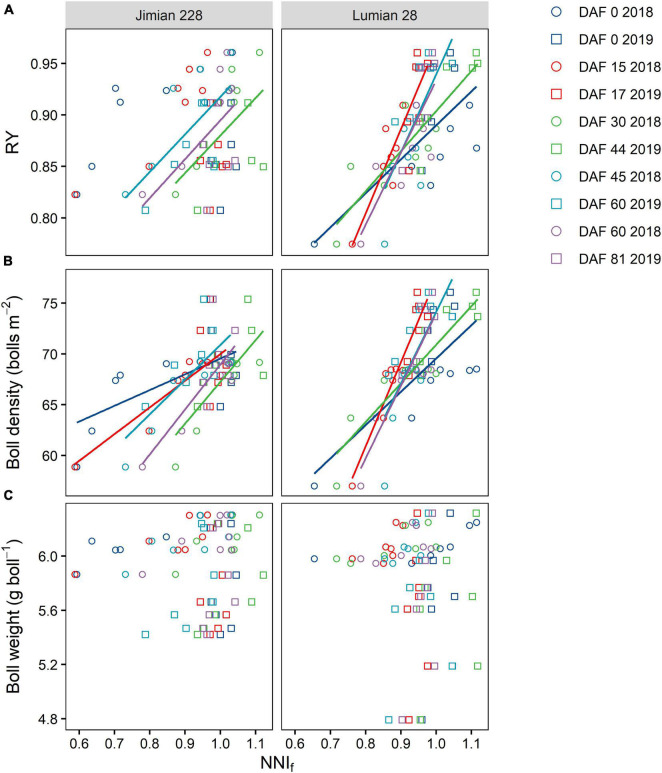
The relationships of RY **(A)**, boll density **(B)**, and boll weight **(C)** with the fruits nitrogen nutrition index (NNI_f_) for each cultivar at each sampling date. RY, relative yield; DAF, days after flowering; and the NNI_f_ is calculated as the ratio between the actual fruits N concentration and the critical fruits N concentration. Calculation for relative yield is presented in section “Materials and Methods.” Different symbols indicate mean values of the three replicate plots. The color solid lines represent significant linear relationships (*P* < 0.05) for Jimian 228 and Lumian 28 at each sampling time across 2 years; non-significant relationships are not shown (*P* > 0.05).

Across the 2-year period, the relationship between RY vs. NNI_f_ was described by a simple linear model under two cultivars at most sampling dates, except for 0 and 17 DAF for Jimian 228 (Figure 7A). However, RY could not reach 1 at an NNI_f_ of approximately 1. When the RY was 0.95, the NNI_f_ reached 1.143 and 1.063 for Jimian 228 and Lumian 28, respectively. Moreover, the responses of yield components to the NNI_f_ showed that boll density was positively correlated with the NNI_f_ in both cultivars (Figure 7B), although this pattern did not repeat in terms of boll weight vs. NNI_f_ (Figure 7C). On average, when the NNI_f_ = 1.0, the boll density reached 69.3 and 73.2 bolls m^–2^ for Jimian 228 and Lumian 28, respectively.

### Petiole NO_3_-N Concentration and Its Relationship With the Nitrogen Nutrition Index of Fruits

**FIGURE 8:**
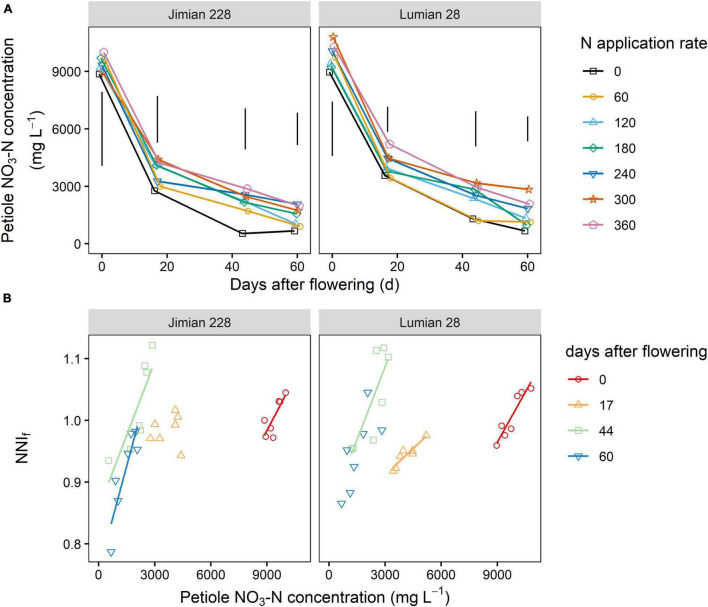
Petiole NO_3_-N concentration **(A)** and its relationship to the fruits nitrogen nutrition index (NNI_f_) **(B)** for Jimian 228 and Lumian 28 in 2019. Data show means of the three replicate plots. The NNI_f_ is calculated as the ratio between the actual fruits N concentration and the critical fruits N concentration. **(A)** The vertical bars indicate Tukey’s HSD error bars at each sampling date. Full model ANOVA results are provided in Supplementary Table 6. **(B)** The color solid lines represent significant linear relationships (*P* < 0.05); non-significant relationships are not shown (*P* > 0.05).

The nitrogen fertilization effect (*P* = 0.0004) was more pronounced than the cultivar effect on the petiole NO_3_-N concentration (*P* = 0.0141) (Supplementary Table 6). N fertilization increased the NO_3_-N concentration but no significant increase beyond 120 kg ha^–1^ was recorded. On average, the petiole NO_3_-N concentration was 4,143 and 4,449 mg L^–1^ for Jimian 228 and Lumian 28, respectively. Moreover, the petiole NO_3_-N concentration of Lumian 28 was consistently greater than that of Jimian 228 during any growth stage and N application rate (Figure 8A and Supplementary Table 6).

Overall, there was a positive relationship between petiole NO_3_-N concentration and the NNI_f_ under the two cotton cultivars, although it was not significant at 17 DAF for Jimian 228 and 60 DAF for Lumian 28 (Figure 8B). This relationship indicated that the NO_3_-N concentration in the petiole was a good predictor of the NNI_f_ and consequently could be an alternative approach to evaluate fruits N status.

## Discussion

### Cotton Growth, Yield, and Nitrogen Responses

In the present study, significant effects on cotton shoot DM, fruits DM and [N] were observed in response to the N application rate. The shoot and fruits DM observed here were 8.88–15.7 and 4.02–8.48 t ha^–1^ for Jimian 228, 6.9–12.43 t ha^–1^ and 4.34–7.72 t ha^–1^ for Lumian 28, respectively, which are comparable to those reported by previous studies, depending on the treatments applied and environmental conditions (Bange and Milroy, 2004; Zhang et al., 2008; Dai et al., 2015). For example, in a similar agro-ecological region (near our study site), aboveground DM was 12.7 t ha^–1^ and fruits DM was 8.0 t ha^–1^ at a high plant density of 10.5 plants m^–2^ (Dai et al., 2015). Bange and Milroy (2004) also reported final shoot dry matter up to 18.7 t ha^–1^ varying significantly between diverse cotton genotypes in New South Wales of Australia. In addition, significant effects of the N fertilizer rate on DMs in shoot and fruits were more pronounced than on the respective [N] in the same sampling date, suggesting that fruits and shoot DMs were more responsive to N fertilizer than the respective [N].

However, a limited cotton yield response to the N fertilizer rate was observed, with a significant cultivar effect, indicating that the effects of the cultivar were far greater than those of N fertilizer. Little or no yield response to fertilizer N application was also reported by Dong et al. (2012) in the Yellow River Delta of China (near our study site) and Stamatiadis et al. (2016) in central Greece. The former observed a yield response to the N fertilizer rate only at low plant density (3 plants m^–2^). Contrary to our results, Li et al. (2017) found a linear increase in N concentration with increasing rates of N fertilizer despite the leveling-off or decline of yield. This conflict is likely derived from the difference between the critical soil N level or tested cultivar sensitivity to N fertilizer (Stamatiadis et al., 2016).

### Critical Nitrogen Dilution Curve for Cotton Fruits

Fruits [N] gradually decreased with increasing fruits DM, due to the N dilution effect resulting from an increase in biomass accumulation that was greater than the N uptake (Lemaire et al., 2007). The N dilution effect has also been widely reported in several crop species, including winter wheat (Justes et al., 1994), maize (Ziadi et al., 2008b) and rice (Ata-Ul-Karim et al., 2013). In this study, the mean value of the [N]_c_ in fruits with DM of 1 t ha^–1^ (2.49), estimated by the coefficient (*a*) of the N dilution curves, was much lower than that in the shoot biomass (greater than 5.0) (Xue et al., 2007; Zhang et al., 2008). This discrepancy may be related to the growth stage (Zhao et al., 2016a). The development stage when cotton fruits was equal to 1 t ha^–1^ is later than that when shoot biomass was 1 t ha^–1^. The dilution coefficient (*b*), however, was similar to that observed previously (Xue et al., 2007; Zhang et al., 2008), suggesting that there was a similar dilution process between fruits and the shoot as a result of the plants’ indeterminate growth habit. However, fruits N is more dependent on physiological N mobilization from stems and leaves, while only N added at fruit formation is directly mobilized to fruits (see below).

The critical N dilution curve for cotton fruits, in our study, had a notably lower dilution of N with increasing dry mass than a universal curve for C3 crops reported by Lemaire and Gastal (1997). The limited N dilution of fruits is partly explained by the indeterminate nature. Cotton plants continuously produce new organs, including fruits, simultaneously, which differs in many respects from C3 crops. Continued production of new fruits in cotton crops maintains a relatively high N content throughout the crop growth, which was attributed to the high translocation of N from stems and leaves to fruits. In other crops, relatively limited dilution was also observed in sunflower (Debaeke et al., 2012) and sweet pepper (Rodríguez et al., 2020).

In addition, fruits-based critical N curves showed small variations between the tested cotton cultivars differing in maturity. A recent study reported that the changes in plant canopy architecture may be a potential interpretation of variations in the N dilution curve (Ciampitti et al., 2021). Therefore, plant architecture, rather than precocity, needs to be considered with a focus on the genotypic effects on the N dilution curve in future studies.

Although the critical N curve developed for cotton fruits could effectively differentiate between N limiting and N non-limiting situations within the range for which it was established, note that this study was conducted in a single field site. Therefore, further study with additional sites will be necessary since the determination of [N] with shoot biomass might be affected by sites as reported for rice (Ata-Ul-Karim et al., 2013) and forage grasses (Agnusdei et al., 2010).

### Nitrogen Nutrition Index

The critical N dilution curve allows for the determination of the NNI, which quantifies the intensity of both N deficiency and N luxury consumption for a given crop (Lemaire and Gastal, 1997). Values of the NNI close to 1 indicate that the crop is not limited by the N supply, while values lower or higher than 1 indicate N deficiency or N luxury consumption, respectively. The NNI_f_ changed little with cotton growth after the flowering period, indicating a stable N requirement in fruits after the flowering period.

The positive relationship between the NNI_f_ and NNI_sh_ under the two cultivars at most sampling dates suggested that the NNI in shoot biomass could be easily estimated using the NNI in fruits. These results were aligned with similar recent findings in which a close relationship between the NNI in vines or tubers and the NNI in total biomass was observed for potato (Giletto et al., 2020). Therefore, N nutrition sufficiency can be estimated with derived NNI_f_ values from fruits based on N dilution curve by only sampling for cotton fruits biomass, which would represent a fast and cost-effective option.

The linear relationship of RY with the NNI_f_ indicated that the RY was nearly 95% when the NNI_f_ was approximately one, and it decreased with a decreasing NNI_f_ below 1. Therefore, our results demonstrated that the NNI_f_ adequately identified the N status in cotton cultivars and had potential as a plant diagnostic tool to estimate the N status of cotton crops.

In this study, the NNI_f_ was used to predict the yield response to N fertilization and to develop strategies to manage crop N nutrition to match the N supply with crop demand after the flowering period. As a result, attention should be paid to the time lag if we use it to guide N fertilizer application in cotton production, especially at the early stage of cotton growth.

### Alternative Diagnostic Methods

Although the NNI can be used to guide N management in crop production, the measurement is a laborious process that greatly limits the practical application of these diagnostic methods. Therefore, a rapid measurement method is needed. A good correlation between petiole NO_3_-N concentration and the NNI_f_ was observed in our study, suggesting that the NNI_f_ could be estimated with petiole NO_3_-N concentration. Moreover, the use of petiole NO_3_-N has the advantages of being a more rapid and simple measurement than determinations of shoot DM and [N] (Wu et al., 2007). Therefore, this indirect method could potentially provide an alternative for estimating the NNI, avoiding time-consuming and labor-intensive shoot biomass sampling, and could characterize crops and environments in situations where the NNI cannot be measured directly.

Future research efforts should attempt to investigate whether other N diagnostic tools (e.g., chlorophyll meters, active crop sensors, and remote sensing) can be used to estimate the N status across different cultivars and environmental conditions (Liu et al., 2020; Shankar et al., 2020), which could complement our results.

## Conclusion

The fruits [N]_c_ decreased with increasing fruits DM in cotton due to the N dilution process. A unique critical fruits N dilution curve of [N]_c_ = 2.49 × DM^–0.12^ (DM, fruits dry mass; *R*^2^ = 0.649, *P* < 0.0001), with [N]_c_ as the critical N concentration in fruits, was developed for cotton fruits across cultivar-years. The fruits N dilution curve could effectively discriminate between N limiting and N non-limiting situations within the range for which it was established. From the dilution curve developed for fruits DM, the NNI_f_ was determined, which can be used as an N status diagnostic tool. The NNI_f_ values were highly related to the NNI_sh_, suggesting that the NNI in shoot biomass could be easily estimated using the NNI in fruits. On average, the RY was nearly 95% for an NNI_f_ at approximately 1, while it decreased with a decreasing NNI_f_ below 1. The NNI_f_ was also linearly related to petiole NO_3_-N concentration, suggesting that NO_3_-N concentration in petioles is a good predictor of the NNI_f_. The critical fruits N dilution curve developed in this study provides insight into plant N nutrition and can serve as a guide to improve N diagnosis and management in cotton production. Future research efforts should attempt to investigate whether the N nutrition index in specific plant organs can be used to determine threshold values for N sufficiency with other N diagnostic tools (e.g., chlorophyll meters, active crop sensors and remote sensing). Moreover, more attention should be paid to the time lag if we use this method to guide N fertilizer application in cotton production, especially at the early stage of cotton growth.

## Data Availability Statement

The raw data supporting the conclusions of this article will be made available by the authors, without undue reservation.

## Author Contributions

HD, PL, and CZ designed the experiment. WF performed the field and lab work with help from YQ, MS, and JS. WF and XL analyzed the data and led the writing of the manuscript. All authors contributed to editing the manuscript and gave final approval for publication.

## Conflict of Interest

The authors declare that the research was conducted in the absence of any commercial or financial relationships that could be construed as a potential conflict of interest.

## Publisher’s Note

All claims expressed in this article are solely those of the authors and do not necessarily represent those of their affiliated organizations, or those of the publisher, the editors and the reviewers. Any product that may be evaluated in this article, or claim that may be made by its manufacturer, is not guaranteed or endorsed by the publisher.

## Abbreviations

[N]_c_: critical nitrogen concentration
DAF: days after flowering
DM: dry mass
N: nitrogen
[N]: nitrogen concentration
NNI: nitrogen nutrition index
NNI_f_: nitrogen nutrition index of fruits
NNI_sh_: nitrogen nutrition index of the shoot
RY: relative yield.
