# Supplementary material for: Fruits-Based Critical Nitrogen Dilution Curve for Diagnosing Nitrogen Status in Cotton
Source: Front Plant Sci. 2022 Jan 28;13:801968. doi: 10.3389/fpls.2022.801968 (PMC8831891; doi:10.3389/fpls.2022.801968)
Supplement: Supplementary file 1 [file Data_Sheet_1.docx]

Supplementary Material

# Supplementary Figures and Tables

## Supplementary Figures


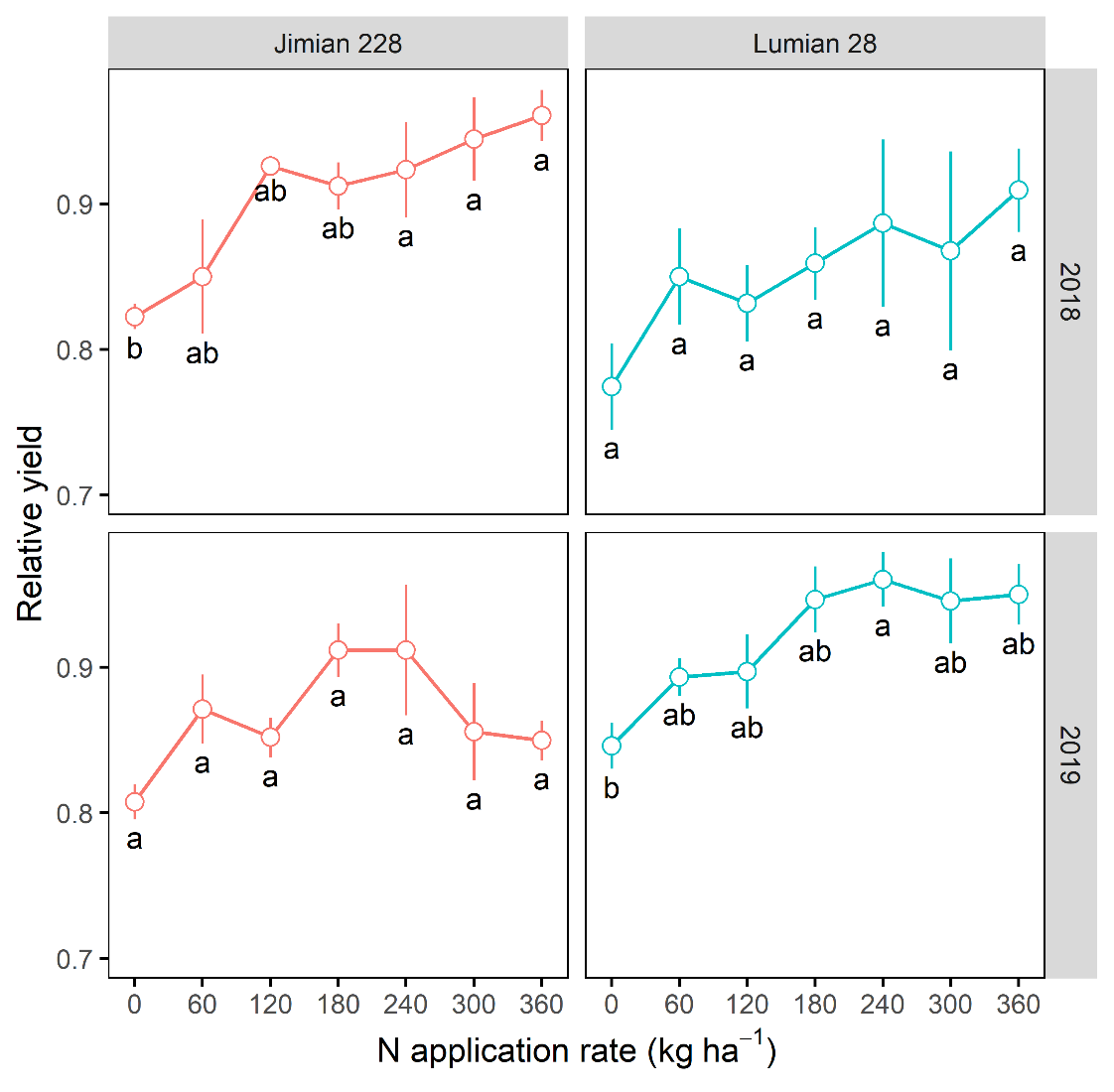


**Supplementary Figure 1.** Relative yield in response to N application rate for Jimian 228 and Lumian 28 in 2018 and 2019. The relative yield is the ratio between the lint yield of each plot and the maximum lint yield of each cultivar in each year. Data show means ± SE (*n* = 3). Different letters indicate significant differences using Tukey’s HSD test at *P* < 0.05.


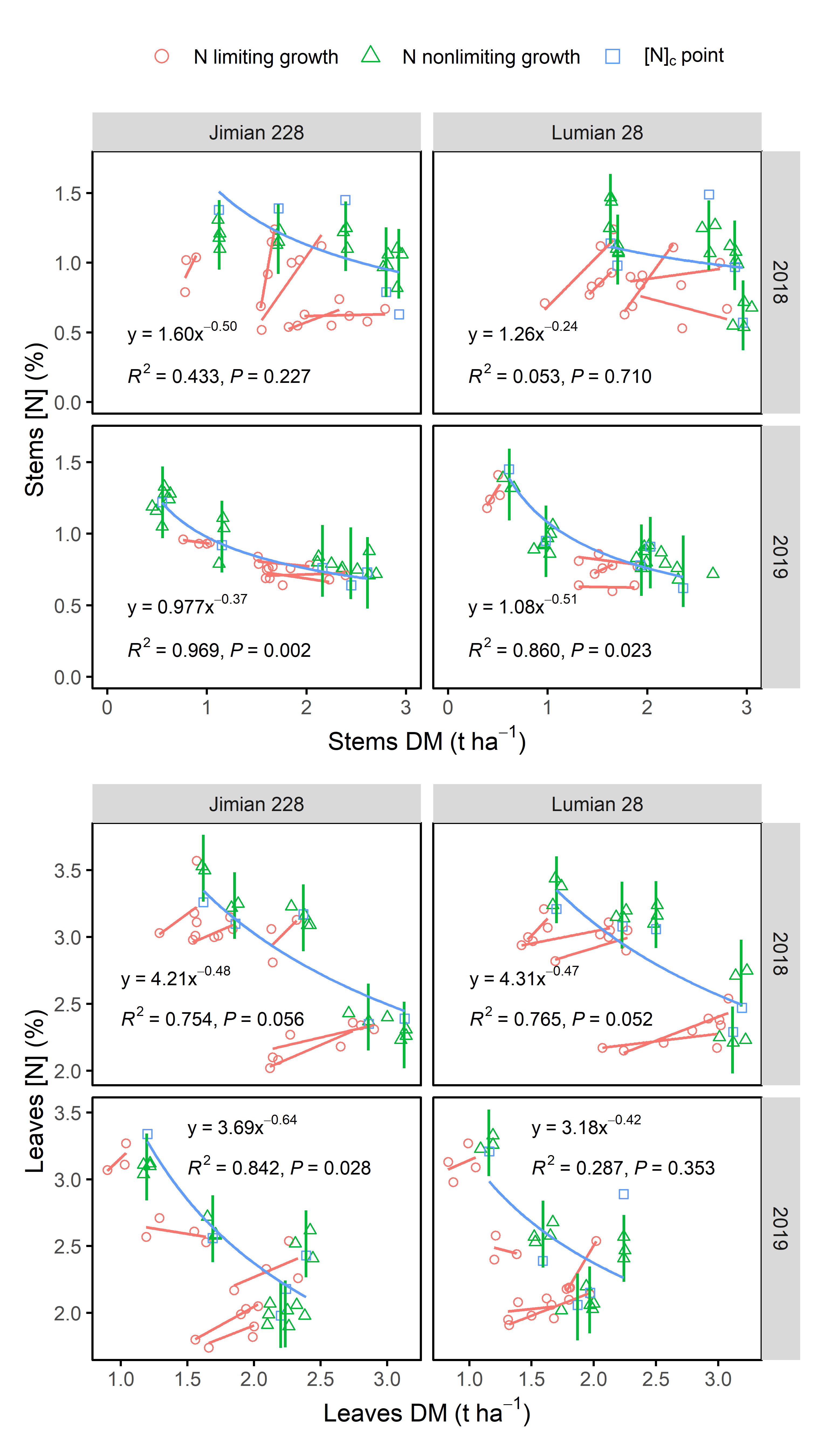


**Supplementary Figure 2.** Critical stems and leaves N dilution curves for Jimian 228 and Lumian 28 in 2018 and 2019. DM, dry mass; [N], N concentration and [N]_c_, critical N concentration. Data show means of the three replicates. The red lines represent simple linear regression of N limiting growth points at each sampling date. The green lines represent the mean values of dry mass by N nonlimiting growth points at each sampling date. The blue lines represent the fitted critical N curves for cotton stems and leaves using [N]_c_ points. For calculations for the stems and leaves critical N dilution points, see **MATERIALS AND METHODS** section.

## Supplementary Tables

**Supplementary Table 1.** Observations of cotton phenological events in 2018–2020. The average dates of emergence, anthesis and maturity of

| Year | Cultivar | Plant emergence | Squaring | Flowering | Boll opening |
| --- | --- | --- | --- | --- | --- |
| 2018 | Jimian 228 | 5 May | 8 June | 5 July | 21 August |
|  | Lumian 28 | 5 May | 7 June | 3 July | 20 August |
| 2019 | Jimian 228 | 7 May | 10 June | 2 July | 25 August |
|  | Lumian 28 | 7 May | 9 June | 1 July | 23 August |
| 2020 | Jimian 228 | 2 May | 12 June | 6 July | 31 August |
|  | Lumian 28 | 2 May | 10 June | 3 July | 28 August |

The dates of each event were recorded when 50% of observed plants in each plot reached it.

**Supplementary Table 2.** Cotton fruits DM, shoot DM, and their N concentrations ([N]) in response to the N application rate for Jimian 228 and Lumian 28 in 2018 and 2019.

| Year | Cultivar | DAF | N applicate rate (kg ha^–1^) | | | | | | | | |
| --- | --- | --- | --- | --- | --- | --- | --- | --- | --- | --- | --- |
|  |  |  | 0 | 60 | 120 | 180 | 240 | 300 | 360 | *P* value | Tukey’s HSD |
|  |  |  | Fruits DM (t ha^–1^) | | | | | | | | |
| 2018 | Jimian 228 | 0 | 0.26 b | 0.33 ab | 0.33 ab | 0.35 ab | 0.35 ab | 0.37 ab | 0.42 a | 0.0098 | 0.104 |
|  |  | 15 | 2.27 d | 3.12 c | 3.89 b | 3.95 b | 4.39 a | 4.69 a | 4.73 a | < 0.0001 | 0.408 |
|  |  | 30 | 3.76 d | 4.74 c | 5.97 ab | 5.84 b | 6.03 ab | 6.30 ab | 6.48 a | < 0.0001 | 0.531 |
|  |  | 45 | 4.40 c | 7.10 b | 8.17 a | 8.34 a | 8.38 a | 8.41 a | 8.52 a | < 0.0001 | 0.789 |
|  |  | 60 | 5.20 c | 6.88 b | 8.01 a | 7.92 a | 8.35 a | 8.37 a | 8.04 a | < 0.0001 | 0.977 |
|  | Lumian 28 | 0 | 0.49 c | 0.56 bc | 0.60 abc | 0.64 ab | 0.68 ab | 0.66 ab | 0.69 a | 0.00101 | 0.123 |
|  |  | 15 | 1.96 b | 2.29 b | 3.21 a | 3.29 a | 3.40 a | 3.39 a | 3.69 a | < 0.0001 | 0.704 |
|  |  | 30 | 2.66 c | 3.34 b | 4.66 a | 5.05 a | 5.15 a | 5.23 a | 5.13 a | < 0.0001 | 0.578 |
|  |  | 45 | 3.80 d | 4.82 c | 6.07 b | 7.61 a | 7.84 a | 7.63 a | 7.83 a | < 0.0001 | 0.778 |
|  |  | 60 | 4.02 c | 5.17 c | 6.44 b | 7.79 a | 8.26 a | 7.85 a | 8.48 a | < 0.0001 | 1.241 |
| 2019 | Jimian 228 | 0 | 0.10 a | 0.11 a | 0.12 a | 0.11 a | 0.10 a | 0.10 a | 0.10 a | 0.6580 | 0.042 |
|  |  | 17 | 0.48 a | 0.57 a | 0.58 a | 0.58 a | 0.61 a | 0.61 a | 0.64 a | 0.5140 | 0.274 |
|  |  | 44 | 2.38 b | 2.90 ab | 3.02 ab | 3.32 ab | 3.54 a | 3.54 a | 3.79 a | 0.0048 | 0.986 |
|  |  | 60 | 4.73 b | 4.83 b | 5.22 ab | 4.89 b | 5.90 a | 6.04 a | 5.99 a | 0.0002 | 0.846 |
|  |  | 81 | 5.18 d | 5.47 cd | 5.92 bcd | 6.26 abcd | 6.90 abc | 7.33 ab | 7.45 a | 0.0009 | 1.513 |
|  | Lumian 28 | 0 | 0.10 a | 0.11 a | 0.13 a | 0.12 a | 0.11 a | 0.13 a | 0.12 a | 0.4540 | 0.054 |
|  |  | 17 | 0.55 a | 0.57 a | 0.67 a | 0.66 a | 0.75 a | 0.69 a | 0.65 a | 0.5030 | 0.352 |
|  |  | 44 | 2.80 b | 2.84 b | 3.24 ab | 3.31 ab | 3.51 a | 3.41 ab | 3.56 a | 0.0069 | 0.657 |
|  |  | 60 | 4.06 b | 4.70 ab | 5.24 a | 5.07 ab | 5.70 a | 5.76 a | 5.78 a | 0.0011 | 1.109 |
|  |  | 81 | 4.34 d | 5.56 c | 6.16 bc | 6.47 bc | 6.98 ab | 7.71 a | 7.72 a | < 0.0001 | 0.992 |
|  |  |  | Shoot DM (t ha^–1^) | | | | | | | | |
| 2018 | Jimian 228 | 0 | 2.53 c | 2.91 b | 3.03 b | 3.29 a | 3.30 a | 3.37 a | 3.40 a | < 0.0001 | 0.214 |
|  |  | 15 | 5.81 d | 6.97 c | 7.88 b | 8.11 b | 8.60 a | 8.94 a | 9.01 a | < 0.0001 | 0.466 |
|  |  | 30 | 8.09 d | 9.46 c | 11.09 b | 11.25 b | 11.77 ab | 12.04 a | 12.08 a | < 0.0001 | 0.693 |
|  |  | 45 | 9.06 d | 12.14 c | 14.19 b | 14.56 ab | 15.34 a | 15.28 ab | 15.25 ab | < 0.0001 | 1.122 |
|  |  | 60 | 10.10 c | 12.57 b | 14.56 a | 14.76 a | 15.63 a | 15.70 a | 15.25 a | < 0.0001 | 1.215 |
|  | Lumian 28 | 0 | 3.18 d | 3.81 c | 4.08 bc | 4.23 ab | 4.35 a | 4.37 a | 4.33 ab | < 0.0001 | 0.266 |
|  |  | 15 | 5.21 c | 6.36 b | 7.57 a | 7.73 a | 8.01 a | 7.96 a | 8.20 a | < 0.0001 | 0.743 |
|  |  | 30 | 6.64 d | 8.08 c | 9.48 b | 10.39 ab | 11.12 a | 11.17 a | 11.19 a | < 0.0001 | 0.934 |
|  |  | 45 | 8.36 d | 10.55 c | 12.80 b | 14.55 a | 14.90 a | 14.92 a | 14.94 a | < 0.0001 | 1.014 |
|  |  | 60 | 8.88 d | 11.18 c | 13.30 b | 15.02 a | 15.52 a | 15.23 a | 15.70 a | < 0.0001 | 1.464 |
| 2019 | Jimian 228 | 0 | 1.45 a | 1.64 a | 1.70 a | 1.85 a | 1.83 a | 1.94 a | 1.95 a | 0.1100 | 0.592 |
|  |  | 17 | 2.42 c | 2.78 bc | 3.22 ab | 3.16 ab | 3.39 ab | 3.48 a | 3.52 a | 0.0008 | 0.674 |
|  |  | 44 | 5.76 c | 6.50 bc | 7.01 abc | 7.60 ab | 7.96 a | 8.24 a | 8.32 a | 0.0002 | 1.406 |
|  |  | 60 | 8.05 c | 8.37 c | 8.88 c | 9.22 bc | 10.58 ab | 10.65 ab | 10.87 a | < 0.0001 | 1.541 |
|  |  | 81 | 8.36 e | 9.00 de | 9.58 cde | 10.56 bcd | 11.43 abc | 11.96 ab | 12.43 a | < 0.0001 | 1.859 |
|  | Lumian 28 | 0 | 1.36 b | 1.36 b | 1.70 ab | 1.60 ab | 1.75 ab | 1.94 ab | 1.97 a | 0.0134 | 0.587 |
|  |  | 17 | 2.61 b | 2.73 ab | 3.01 ab | 3.01 ab | 3.32 a | 3.35 a | 3.35 a | 0.0051 | 0.625 |
|  |  | 44 | 6.07 c | 6.16 c | 6.69 bc | 7.32 ab | 7.73 a | 7.79 a | 7.82 a | < 0.0001 | 0.864 |
|  |  | 60 | 6.69 c | 7.70 bc | 8.94 ab | 9.01 ab | 9.61 a | 9.66 a | 9.65 a | 0.0003 | 1.708 |
|  |  | 81 | 6.90 e | 8.54 d | 9.62 cd | 10.30 bc | 10.89 abc | 11.55 ab | 12.28 a | < 0.0001 | 1.464 |
|  |  |  | Fruits [N] (%) | | | | | | | | |
| 2018 | Jimian 228 | 0 | 2.32 d | 2.39 d | 2.63 cd | 2.65 cd | 3.14 bc | 3.46 ab | 3.71 a | < 0.0001 | 0.513 |
|  |  | 15 | 2.24 a | 2.24 a | 2.37 a | 2.49 a | 2.36 a | 2.29 a | 2.46 a | 0.4640 | 0.491 |
|  |  | 30 | 1.80 b | 1.97 ab | 2.19 a | 2.13 a | 2.17 a | 2.09 a | 2.12 a | 0.0056 | 0.283 |
|  |  | 45 | 1.71 a | 1.74 a | 1.75 a | 1.73 a | 1.70 a | 1.75 a | 1.79 a | 0.9390 | 0.277 |
|  |  | 60 | 1.86 a | 1.81 a | 1.91 a | 1.84 a | 1.80 a | 1.87 a | 1.88 a | 0.4720 | 0.195 |
|  | Lumian 28 | 0 | 2.16 c | 2.82 b | 3.06 a | 3.09 a | 3.21 a | 3.11 a | 3.25 a | < 0.0001 | 0.216 |
|  |  | 15 | 1.90 c | 2.01 bc | 1.99 bc | 2.18 ab | 2.32 a | 2.14 ab | 2.28 a | 0.0002 | 0.230 |
|  |  | 30 | 1.58 b | 2.12 a | 2.06 ab | 2.08 a | 2.12 a | 2.27 a | 2.24 a | 0.0075 | 0.497 |
|  |  | 45 | 1.58 b | 1.57 b | 1.67 ab | 1.75 ab | 1.84 a | 1.81 ab | 1.68 ab | 0.0116 | 0.241 |
|  |  | 60 | 1.71 b | 1.69 b | 1.74 ab | 1.82 a | 1.75 ab | 1.72 b | 1.70 b | 0.0023 | 0.078 |
| 2019 | Jimian 228 | 0 | 2.99 a | 3.03 a | 2.89 a | 3.04 a | 2.89 a | 2.89 a | 3.11 a | 0.3047 | 0.375 |
|  |  | 17 | 2.45 a | 2.58 a | 2.50 a | 2.44 a | 2.38 a | 2.30 a | 2.45 a | 0.6110 | 0.502 |
|  |  | 44 | 2.21 a | 1.97 a | 2.03 a | 2.03 a | 2.19 a | 2.21 a | 2.26 a | 0.1120 | 0.373 |
|  |  | 60 | 1.55 b | 1.77 ab | 1.69 ab | 1.85 a | 1.76 ab | 1.87 a | 1.78 ab | 0.0377 | 0.299 |
|  |  | 81 | 1.87 a | 1.84 a | 1.86 a | 1.90 a | 1.85 a | 1.95 a | 1.91 a | 0.9070 | 0.340 |
|  | Lumian 28 | 0 | 2.86 a | 2.92 a | 2.92 a | 2.89 a | 3.06 a | 3.05 a | 3.03 a | 0.0573 | 0.242 |
|  |  | 17 | 2.29 a | 2.27 a | 2.44 a | 2.26 a | 2.27 a | 2.30 a | 2.20 a | 0.5300 | 0.390 |
|  |  | 44 | 1.99 b | 2.08 ab | 2.12 ab | 2.10 ab | 2.26 a | 2.24 a | 2.26 a | 0.0065 | 0.228 |
|  |  | 60 | 1.73 a | 1.74 a | 1.80 a | 1.86 a | 1.88 a | 1.89 a | 2.01 a | 0.1780 | 0.361 |
|  |  | 81 | 1.79 a | 1.82 a | 1.86 a | 1.78 a | 1.87 a | 1.78 a | 1.86 a | 0.8860 | 0.320 |
|  |  |  | Shoot [N] (%) | | | | | | | | |
| 2018 | Jimian 228 | 0 | 2.20 d | 2.34 c | 2.39 c | 2.34 c | 2.64 b | 2.66 b | 2.77 a | < 0.0001 | 0.104 |
|  |  | 15 | 2.00 b | 2.12 ab | 2.25 ab | 2.35 a | 2.28 ab | 2.27 ab | 2.37 a | 0.0124 | 0.303 |
|  |  | 30 | 1.83 b | 2.03 a | 2.18 a | 2.15 a | 2.14 a | 2.13 a | 2.16 a | 0.0001 | 0.169 |
|  |  | 45 | 1.54 b | 1.60 ab | 1.63 ab | 1.69 ab | 1.70 ab | 1.72 a | 1.77 a | 0.0090 | 0.176 |
|  |  | 60 | 1.65 b | 1.65 b | 1.74 ab | 1.70 ab | 1.71 ab | 1.79 a | 1.80 a | 0.0072 | 0.132 |
|  | Lumian 28 | 0 | 2.09 c | 2.07 c | 2.28 bc | 2.35 ab | 2.58 a | 2.48 ab | 2.52 ab | < 0.0001 | 0.242 |
|  |  | 15 | 1.87 c | 2.07 b | 2.07 b | 2.19 ab | 2.30 a | 2.21 ab | 2.27 a | < 0.0001 | 0.150 |
|  |  | 30 | 1.64 b | 2.00 a | 2.03 a | 2.08 a | 2.09 a | 2.23 a | 2.23 a | 0.0010 | 0.341 |
|  |  | 45 | 1.57 bc | 1.57 c | 1.64 abc | 1.73 ab | 1.77 a | 1.73 ab | 1.68 abc | 0.0042 | 0.164 |
|  |  | 60 | 1.62 b | 1.59 ab | 1.65 ab | 1.69 ab | 1.71 a | 1.70 ab | 1.69 ab | 0.0178 | 0.111 |
| 2019 | Jimian 228 | 0 | 2.48 a | 2.52 a | 2.54 a | 2.52 a | 2.53 a | 2.50 a | 2.52 a | 0.9940 | 0.307 |
|  |  | 17 | 2.05 a | 2.09 a | 2.02 a | 2.04 a | 2.01 a | 2.05 a | 2.04 a | 0.9780 | 0.289 |
|  |  | 44 | 1.82 a | 1.82 a | 1.81 a | 1.84 a | 1.92 a | 1.88 a | 1.99 a | 0.4220 | 0.316 |
|  |  | 60 | 1.38 b | 1.63 a | 1.60 a | 1.60 a | 1.60 a | 1.63 a | 1.61 a | 0.0009 | 0.154 |
|  |  | 81 | 1.63 a | 1.63 a | 1.67 a | 1.63 a | 1.65 a | 1.68 a | 1.67 a | 0.9589 | 0.231 |
|  | Lumian 28 | 0 | 2.45 a | 2.53 a | 2.52 a | 2.67 a | 2.63 a | 2.63 a | 2.65 a | 0.2500 | 0.329 |
|  |  | 17 | 1.88 a | 1.93 a | 1.95 a | 1.98 a | 2.00 a | 2.00 a | 2.06 a | 0.6380 | 0.344 |
|  |  | 44 | 1.74 a | 1.78 a | 1.80 a | 1.87 a | 1.97 a | 1.96 a | 1.96 a | 0.1040 | 0.320 |
|  |  | 60 | 1.58 a | 1.61 a | 1.64 a | 1.68 a | 1.75 a | 1.70 a | 1.78 a | 0.2120 | 0.280 |
|  |  | 81 | 1.61 a | 1.63 a | 1.66 a | 1.60 a | 1.63 a | 1.63 a | 1.65 a | 0.9810 | 0.235 |

DAF, days after flowering. Shown are the means of the three replicates and the value of Tukey’s HSD error bar. Values followed by different letters within a row are significantly different using Tukey’s HSD (*P* < 0.05). Full model ANOVA results are provided in **Supplementary Table 3**.

**Supplementary Table 3.** ANOVA table for cotton fruits DM, shoot DM and their N concentrations from mixed effects models with sampling date (D), year (Y), N rate (N) and cultivar (C) treated as fixed effects and N rate nested within replicate treated as a random effect.

| Source | Shoot DM | Fruits DM | Fruits [N] | Shoot [N] |
| --- | --- | --- | --- | --- |
| D | < 0.0001 | < 0.0001 | < 0.0001 | < 0.0001 |
| Y | < 0.0001 | < 0.0001 | < 0.0001 | < 0.0001 |
| N | < 0.0001 | < 0.0001 | < 0.0001 | < 0.0001 |
| C | < 0.0001 | < 0.0001 | 0.0002 | 0.0033 |
| D x Y | < 0.0001 | < 0.0001 | 0.1836 | < 0.0001 |
| D x N | < 0.0001 | < 0.0001 | < 0.0001 | 0.0002 |
| D x C | < 0.0001 | < 0.0001 | < 0.0001 | < 0.0001 |
| Y x N | < 0.0001 | < 0.0001 | < 0.0001 | < 0.0001 |
| Y x C | 0.3622 | < 0.0001 | 0.0774 | < 0.0001 |
| N x C | 0.0076 | 0.0001 | 0.0046 | 0.0389 |
| D x Y x N | < 0.0001 | < 0.0001 | < 0.0001 | < 0.0001 |
| D x Y x C | < 0.0001 | < 0.0001 | 0.1020 | 0.0059 |
| D x N x C | 0.0644 | < 0.0001 | 0.0008 | 0.5088 |
| Y x N x C | 0.0034 | < 0.0001 | 0.0399 | 0.8112 |
| D x Y x N x C | 0.0809 | 0.0003 | < 0.0001 | 0.7168 |

DM, dry mass and [N], nitrogen concentration.

**Supplementary Table 4.** Cotton fruits dry mass (DM) and N concentrations ([N]) in response to the N application rate for Jimian 228 and Lumian 28 in 2020.

| Cultivar | DAF | N application rate (kg ha^–1^) | | | | | | | | |
| --- | --- | --- | --- | --- | --- | --- | --- | --- | --- | --- |
|  |  | 0 | 60 | 120 | 180 | 240 | 300 | 360 | *P* value | Tukey’s HSD |
|  |  | Fruits DM (t ha^–1^) | | | | | | | | |
| Jimian 228 | 35 | 1.31 b | 1.82 ab | 2.00 ab | 2.40 a | 2.24 a | 2.16 a | 2.04 ab | 0.0105 | 0.804 |
|  | 60 | 3.57 b | 4.29 ab | 4.30 ab | 4.79 ab | 5.59 a | 5.43 a | 5.16 a | 0.0029 | 1.394 |
| Lumian 28 | 35 | 1.85 c | 1.97 bc | 2.03 abc | 2.42 abc | 2.60 a | 2.47 ab | 2.39 abc | 0.0034 | 0.576 |
|  | 60 | 3.38 c | 3.80 c | 3.89 bc | 5.03 ab | 5.15 a | 5.17 a | 5.19 a | 0.0004 | 1.214 |
|  |  | Fruits [N] (%) | | | | | | | | |
| Jimian 228 | 35 | 2.17 c | 2.25 c | 2.35 bc | 2.62 ab | 2.64 a | 2.65 a | 2.57 ab | < 0.0001 | 0.270 |
|  | 60 | 2.09 a | 1.96 a | 2.14 a | 2.13 a | 2.18 a | 2.21 a | 2.17 a | 0.0846 | 0.264 |
| Lumian 28 | 35 | 2.08 b | 2.21 b | 2.26 b | 2.53 a | 2.56 a | 2.56 a | 2.60 a | < 0.0001 | 0.217 |
|  | 60 | 1.92 a | 2.09 a | 2.18 a | 2.24 a | 2.26 a | 2.24 a | 2.29 a | 0.0736 | 0.397 |
| ANOVA |  | Fruits DM | |  | Fruits [N] | |  |  |  |  |
|  | D | < 0.0001  < 0.0001  0.8315  0.0010  0.0062  0.7796  0.6898 | |  | < 0.0001  0.0001  0.7527  0.0011  0.0080  0.2523  0.6805 | |  |  |  |  |
|  | N |  |  |  |  |  |  |  |  |  |
|  | C |  |  |  |  |  |  |  |  |  |
|  | D x N |  |  |  |  |  |  |  |  |  |
|  | D x C |  |  |  |  |  |  |  |  |  |
|  | C x N |  |  |  |  |  |  |  |  |  |
|  | D x C x N |  |  |  |  |  |  |  |  |  |

DAF, days after flowering. Shown are the means of the three replicates and the value of Tukey’s HSD error bar. Values followed by different letters within a row are significantly different using Tukey’s HSD (*P* < 0.05). *P* values are from linear mixed effects models with sampling date (D), N rate (N) and cultivar (C) treated as fixed effects and N rate nested within replicate treated as a random effect.

**Supplementary Table 5.** ANOVA table for the nitrogen nutrition index of cotton fruits (NNI_f_) at each sampling date after flowering from mixed effects models with year (Y), N rate (N) and cultivar (C) treated as fixed effects and N rate nested within replicate treated as a random effect.

| Cultivar | Days after flowering (d) | | | | | |
| --- | --- | --- | --- | --- | --- | --- |
|  | 0(0) | 15(17) | 30(44) | 45(60) | 60(81) | Overall |
| Y | < 0.0001 | < 0.0001 | < 0.0001 | 0.0480 | 0.0005 | < 0.0001 |
| N | < 0.0001 | 0.0005 | < 0.0001 | < 0.0001 | 0.0001 | < 0.0001 |
| C | < 0.0001 | 0.1129 | < 0.0001 | 0.0849 | 0.0003 | 0.9958 |
| Y × N | < 0.0001 | 0.0001 | 0.0524 | 0.6501 | 0.0017 | < 0.0001 |
| Y × C | < 0.0001 | 0.0738 | < 0.0001 | 0.2599 | 0.1567 | 0.6189 |
| N × C | 0.0270 | 0.1589 | 0.1356 | 0.0072 | 0.7536 | 0.7298 |
| Y × N × C | 0.0055 | 0.0273 | 0.6761 | 0.0233 | 0.2226 | 0.1706 |

The numbers without and with brackets are the days after flowering corresponding to sampling dates in 2018 and 2019, respectively.

**Supplementary Table 6.** ANOVA table for petiole NO_3_-N concentration from mixed effects models with sampling date (D), N rate (N) and cultivar (C) treated as fixed effects and N rate nested within replicate treated as a random effect.

| Source | *F* | *P* | Source | *F* | *P* |
| --- | --- | --- | --- | --- | --- |
| D | 898.792 | < 0.0001 | D x N | 0.711 | 0.7924 |
| N | 10.472 | 0.0004 | D x C | 0.333 | 0.8016 |
| C | 6.249 | 0.0141 | N x C | 1.180 | 0.3234 |
|  |  |  | D x N x C | 0.699 | 0.8054 |
